# Electrophysiological Changes in Children with Isolated Persistent Left Superior Vena Cava: A Case–Control Study

**DOI:** 10.3390/jcm15166494

**Published:** 2026-08-21

**Authors:** Sule Arici, Ozlem Surekli Karakus, Gulperi Yagar Keskin, Erkan Tas, Fatih Alparslan Genc, Sezin Bayraktar, Metin Sungur, Ayse Inci Yildirim

**Affiliations:** Department of Pediatric Cardiology, Koşuyolu High Specialization Training and Research Hospital, Denizer Cad. Cevizli Kavşağı No: 2, Cevizli, Kartal, Istanbul 34865, Turkey; docozlem@gmail.com (O.S.K.); gulperiyagar@hotmail.com (G.Y.K.); etas44@gmail.com (E.T.); fagenc61@gmail.com (F.A.G.); sezinbayraktar@hotmail.com (S.B.); metsungur@yahoo.com (M.S.); ayseinciyildirim@gmail.com (A.I.Y.)

**Keywords:** persistent left superior vena cava, coronary sinus, electrocardiography, arrhythmia, pediatric cardiology

## Abstract

**Background:** Persistent left superior vena cava (PLSVC) is the most common congenital anomaly of the thoracic venous system and is generally considered a benign variant. This study aimed to evaluate arrhythmia-related electrocardiographic parameters in children with isolated PLSVC and to assess the relationship with coronary sinus dimensions. **Methods:** This retrospective cross-sectional study included 26 children with isolated PLSVC and 26 age- and sex-matched healthy controls. All participants underwent transthoracic echocardiography and electrocardiographic analysis. Coronary sinus diameters were measured and indexed to body surface area. Electrocardiographic parameters reflecting atrial conduction and ventricular repolarization heterogeneity, including P-wave dispersion, QTc dispersion, Tp–e interval, and indices of cardiac electrophysiological balance (QT/QRS and QTc/QRS), were evaluated. **Results:** P-wave dispersion (62.15 ± 14.78 vs. 35.59 ± 10.02 ms, *p* < 0.001), QTc dispersion (99.66 ± 30.10 vs. 40.45 ± 10.78 ms, *p* < 0.001), and Tp–e dispersion (67.29 ± 19.10 vs. 38.42 ± 7.13 ms, *p* < 0.001) were significantly higher in the PLSVC group. QT/QRS (4.33 ± 0.59 vs. 3.78 ± 0.36, *p* < 0.001) and QTc/QRS (5.48 ± 0.91 vs. 4.61 ± 0.36, *p* < 0.001) ratios were also increased. Coronary sinus measurements showed weak and inconsistent correlations with electrocardiographic parameters. **Conclusions:** Children with isolated PLSVC exhibit significant electrocardiographic alterations reflecting atrial and ventricular electrical heterogeneity. These findings suggest that PLSVC may be associated with subclinical electrophysiological abnormalities beyond a benign anatomical variant. Accordingly, increasing clinical awareness of potential arrhythmic risk during follow-up and considering this aspect in patient evaluation may be appropriate.

## 1. Introduction

Persistent left superior vena cava (PLSVC) is the most common congenital anomaly of the thoracic venous system, with a reported prevalence of approximately 0.3–0.5% in the general population and a higher frequency among patients with congenital heart disease [1,2]. In most cases, PLSVC drains into the right atrium via the coronary sinus and does not result in significant hemodynamic compromise. Accordingly, it is often considered an incidental anatomical variant, particularly when identified in isolation without associated structural cardiac abnormalities [1,3].

Despite its generally benign characterization, the anatomical features of PLSVC may have electrophysiological relevance. Coronary sinus dilatation, a characteristic finding in PLSVC, may influence atrial activation and conduction properties due to its close anatomical relationship with the atrial myocardium and conduction system. Previous studies have reported alterations in P-wave morphology and frontal P-wave axis in patients with PLSVC, suggesting that left-sided venous drainage may affect atrial electrical activity [2].

However, current evidence is largely derived from adult populations or selected interventional cohorts, and data regarding children with isolated PLSVC remain limited [2,4,5]. In particular, comprehensive evaluation of noninvasive electrocardiographic markers reflecting atrial conduction and ventricular repolarization heterogeneity has not been adequately addressed in this population.

Therefore, the present study aimed to compare arrhythmia-related electrocardiographic parameters in children with isolated PLSVC and age- and sex-matched healthy controls, and to investigate the relationship between coronary sinus dimensions and these parameters.

## 2. Methods

### 2.1. Study Design and Population

This retrospective, observational, cross-sectional study was conducted at a tertiary pediatric cardiology center.

A total of 52 children were enrolled: 26 patients with isolated PLSVC and 26 age- and sex-matched healthy controls. Patients were identified through retrospective review of institutional electronic medical records between July 2024 and July 2025.

The diagnosis of isolated PLSVC was established based on transthoracic echocardiographic findings, specifically the visualization of a dilated coronary sinus draining into the right atrium, without evidence of additional structural congenital heart disease.

Inclusion criteria for the PLSVC group were age between 0 and 18 years, echocardiographic confirmation of isolated PLSVC, and availability of complete and analyzable 12-lead electrocardiography (ECG) and echocardiographic data.

The control group consisted of healthy children without known structural heart disease, systemic illness, electrolyte imbalance, or medication use affecting cardiac conduction, matched for age and sex.

Exclusion criteria for both groups included incomplete or poor-quality ECG or echocardiographic recordings, presence of additional congenital or acquired heart disease, history of cardiac surgery or interventional procedures, presence of systemic, metabolic, genetic, or neuromuscular disorders, acute clinical conditions (such as fever, infection, or electrolyte imbalance) at the time of ECG acquisition, and use of medications known to affect cardiac electrophysiological parameters.

### 2.2. Echocardiographic Evaluation

Transthoracic echocardiographic examinations were performed in all participants by the same pediatric cardiologist using a Philips EPIQ CVx 3D ultrasound system (Philips Ultrasound LLC, Bothell, WA, USA). Standard two-dimensional, M-mode, and dedicated coronary sinus imaging were obtained in accordance with the recommendations of the American Society of Echocardiography (ASE).

Conventional echocardiographic measurements included interventricular septal thickness (IVSD), left ventricular end-diastolic diameter (LVDD), left ventricular end-systolic diameter (LVSD), left ventricular posterior wall thickness (LVPWD), ejection fraction (EF), and fractional shortening (FS). Z-scores for IVSD, LVDD, LVSD, and LVPWD were calculated using the Boston Children’s Hospital Z-Score Calculator (https://zscore.chboston.org (accessed on 18 August 2026)), based on body surface area-adjusted pediatric reference data. Left ventricular systolic function was assessed by measuring ejection fraction using M-mode echocardiography obtained from the parasternal long-axis view. All measurements were performed during quiet respiration and averaged over three consecutive cardiac cycles.

The diagnosis of PLSVC was confirmed by identifying a dilated coronary sinus draining into the right atrium.

**Figure 1:**
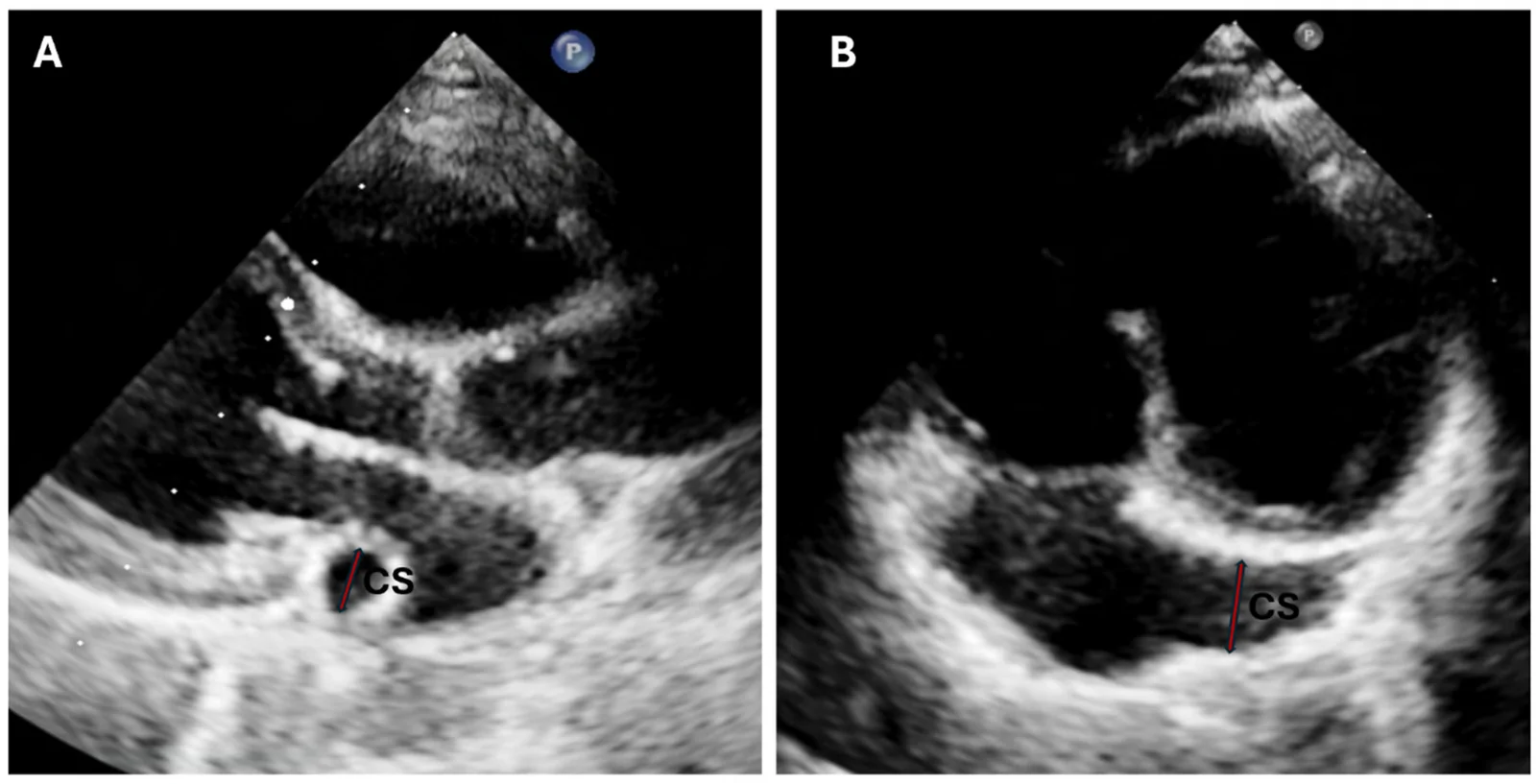
Echocardiographic visualization of the coronary sinus in a patient with persistent left superior vena cava (PLSVC). (**A**). Parasternal long-axis view demonstrating measurement of the coronary sinus diameter. (**B**). Apical four-chamber view with posterior tilt demonstrating measurement of the coronary sinus diameter. The arrows indicate the coronary sinus diameter measurement to the figure caption.

Coronary sinus (CS) measurements were obtained from the apical four-chamber (A4C) view with posterior tilt. In this position, CS diameter was measured approximately 4 mm proximal to its entry into the right atrium. In the parasternal long-axis (PLAX) view, CS diameter was measured as the maximal anteroposterior diameter of the dilated coronary sinus located posterior to the posterior mitral leaflet (Figure 1). All CS measurements were indexed to body surface area (mm/m^2^) to account for inter-individual variability.

### 2.3. Electrocardiographic Analysis

Standard 12-lead ECGs were recorded using a MAC 2000 electrocardiograph (GE Medical Systems Information Technologies, Inc., Milwaukee, WI, USA) at a paper speed of 25 mm/s and a calibration of 10 mm/mV and were analyzed in digital format.

All ECG tracings were exported and magnified to 400% using Adobe Photoshop 2025 (version 26.11.7; Adobe Inc., San Jose, CA, USA) to ensure precise measurement of time intervals. Measurements were performed manually using digital calipers on magnified ECG images.

The following electrocardiographic parameters were evaluated: heart rate and rhythm; P-wave, QRS, and T-wave axes; QRS duration; P-wave dispersion; QT interval and corrected QT (QTc); QTc dispersion; Tp–e interval; Tp–e dispersion; Tp–e/QT and Tp–e/QTc ratios; and QT/QRS and QTc/QRS ratios, representing indices of cardiac electrophysiological balance (ICEB and ICEBc). Corrected QT (QTc) was calculated using Bazett’s formula (QTc = QT/√RR), which was selected because of its widespread use in pediatric electrocardiographic practice and to facilitate comparability with previous studies.

To minimize measurement bias, all ECG parameters were evaluated by an experienced pediatric cardiologist blinded to group allocation.

### 2.4. Ethical Approval

Ethical approval was obtained from the institutional ethics committee (approval number: 2025/16/1224; approval date: 30 September 2025). The study was conducted in accordance with the principles of the Declaration of Helsinki.

Due to the retrospective design of the study, the requirement for informed consent was waived by the ethics committee.

### 2.5. Statistical Analysis

Primary analyses were performed using IBM SPSS Statistics version 22.0; reviewer-requested supplementary calculations used Python 3.13.5. Distributional assumptions were examined before analysis, including with the Shapiro–Wilk test. Continuous variables were summarized as mean ± SD or median (minimum–maximum), and categorical variables as *n* (%). Original SPSS tests and *p* values were retained: independent-samples t, Mann–Whitney U, chi-square/Fisher exact, and Spearman correlation tests were used as appropriate. Additional estimates comprised mean differences with 95% CIs and Hedges’ g for *t*-test variables, Hodges–Lehmann estimates with 10,000-resample bootstrap 95% CIs (seed = 20260810) and Cliff’s delta for Mann–Whitney variables, and Newcombe 95% CIs for risk differences. Fixed-sample power was assessed for *n* = 26 per group. Sensitivity analysis excluded four low atrial rhythm cases while retaining the same analysis approach as in the primary analysis. Interobserver and intraobserver reliability were evaluated with absolute-agreement, single-measure ICC(2,1) and ICC(A,1), respectively, with 95% CIs. All tests were two-sided, and a *p*-value < 0.05 was considered statistically significant.

## 3. Results

### 3.1. Power and Primary Group Comparisons

With 26 participants in each group and a two-sided alpha level of 0.05, the fixed-sample sensitivity analysis showed 80.7% power to detect a standardized effect of Cohen’s d = 0.80 and 94.2% power to detect d = 1.00. The minimum detectable effect was d = 0.792 for 80% power. Thus, the study was adequately powered for large between-group effects but had limited sensitivity for small or moderate effects (Supplementary Table S1).

### 3.2. Demographic and Clinical Characteristics

A total of 52 children were enrolled, including 26 patients with isolated persistent left superior vena cava (PLSVC) and 26 age- and sex-matched healthy controls. The demographic and clinical characteristics of the study population are summarized in Table 1. The mean age was 9.37 ± 5.10 years in the PLSVC group and 9.27 ± 4.95 years in the control group (*p* = 0.943). Sex distribution was identical between groups (female 57.7% and male 42.3% in both; *p* = 1.000). Anthropometric parameters, including height, weight, and body surface area, were comparable between groups (all *p* > 0.05). Likewise, height and weight Z-scores did not differ significantly (all *p* > 0.05).

In the PLSVC group, the most common reason for initial presentation was incidental/asymptomatic detection (69.2%), followed by nonspecific chest pain (15.4%) and arrhythmia-related symptoms (15.4%).

### 3.3. Echocardiographic Findings

Conventional echocardiographic parameters are summarized in Table 2. IVSD was 6.28 ± 1.29 mm in the PLSVC group and 6.28 ± 1.08 mm in controls (*p* = 0.653). LVDD measured 39.11 ± 7.71 mm in the PLSVC group and 38.73 ± 6.95 mm in controls (*p* = 0.852). LVSD was 24.10 ± 4.55 mm in the PLSVC group and 24.50 ± 5.00 mm in controls (*p* = 0.764). LVPWD was 5.89 ± 1.31 mm and 5.62 ± 0.96 mm, respectively (*p* = 0.408).

IVSD Z-score was −0.958 ± 0.854 in the PLSVC group and −0.939 ± 0.747 in controls (*p* = 0.819). LVDD Z-score was −0.255 ± 1.255 versus −0.358 ± 0.767 (*p* = 0.956), and LVSD Z-score was −0.505 ± 1.140 versus −0.396 ± 0.919 (*p* = 0.546). LVPWD Z-score was −1.105 ± 0.852 in the PLSVC group and −1.350 ± 0.482 in controls (*p* = 0.694).

EF was 68.62 ± 4.80% in the PLSVC group and 67.58 ± 5.46% in controls (*p* = 0.470). FS was 37.50 ± 4.62% in the PLSVC group and 37.15 ± 4.14% in controls (*p* = 0.733).

All patients in the PLSVC group had isolated PLSVC draining into the right atrium via the coronary sinus, and no additional structural congenital cardiac anomalies were identified on echocardiographic evaluation.

### 3.4. Coronary Sinus Dimensions

CS measurements are summarized in Table 3. In the apical four-chamber view, CS diameter was 9.01 ± 2.30 mm in the PLSVC group and 3.42 ± 0.99 mm in controls (*p* < 0.001). Indexed CS diameter (mm/m^2^) was 9.50 ± 3.39 in the PLSVC group and 3.57 ± 1.29 in controls (*p* < 0.001).

In the parasternal long-axis view, CS diameter in the PLSVC group was 8.73 ± 2.12 mm, and indexed CS diameter was 9.26 ± 3.31 mm/m^2^. Coronary sinus measurements in this view were obtained only in the PLSVC group, as the coronary sinus could not be consistently visualized or measured in the control group.

### 3.5. Electrocardiographic Parameters

Electrocardiographic parameters are summarized in Table 4. Heart rate did not differ significantly between the groups (98.69 ± 25.32 bpm in the PLSVC group vs. 93.15 ± 20.71 bpm in controls, *p* = 0.392). P-wave axis (29.72 ± 24.22° vs. 37.39 ± 14.24°, *p* = 0.172), QRS axis (53.79 ± 28.46° vs. 58.35 ± 15.58°, *p* = 0.498), and T-wave axis (38.89 ± 24.59° vs. 40.50 ± 12.46°, *p* = 0.498) were also similar between groups.

Sinus rhythm was present in 22 patients (84.6%) in the PLSVC group and in all controls (100%), whereas low atrial rhythm was observed in four patients (15.4%) in the PLSVC group (*p* = 0.110).

*p*-wave dispersion was significantly higher in the PLSVC group compared with controls (62.15 ± 14.78 ms vs. 35.59 ± 10.02 ms, *p* < 0.001). QTc dispersion was also significantly greater in the PLSVC group (99.66 ± 30.10 ms vs. 40.45 ± 10.78 ms, *p* < 0.001). Similarly, Tp–e dispersion was higher in the PLSVC group compared with the control group (67.29 ± 19.10 ms vs. 38.42 ± 7.13 ms, *p* < 0.001).

The Tp–e/QT ratio was higher in the PLSVC group (0.237 ± 0.029 vs. 0.218 ± 0.024, *p* = 0.013), whereas the Tp–e/QTc ratio did not differ significantly between the groups (*p* = 0.195).

The QT/QRS ratio (4.33 ± 0.59 vs. 3.78 ± 0.36, *p* < 0.001) and QTc/QRS ratio (5.48 ± 0.91 vs. 4.61 ± 0.36, *p* < 0.001) were significantly higher in the PLSVC group.

**Figure 2:**
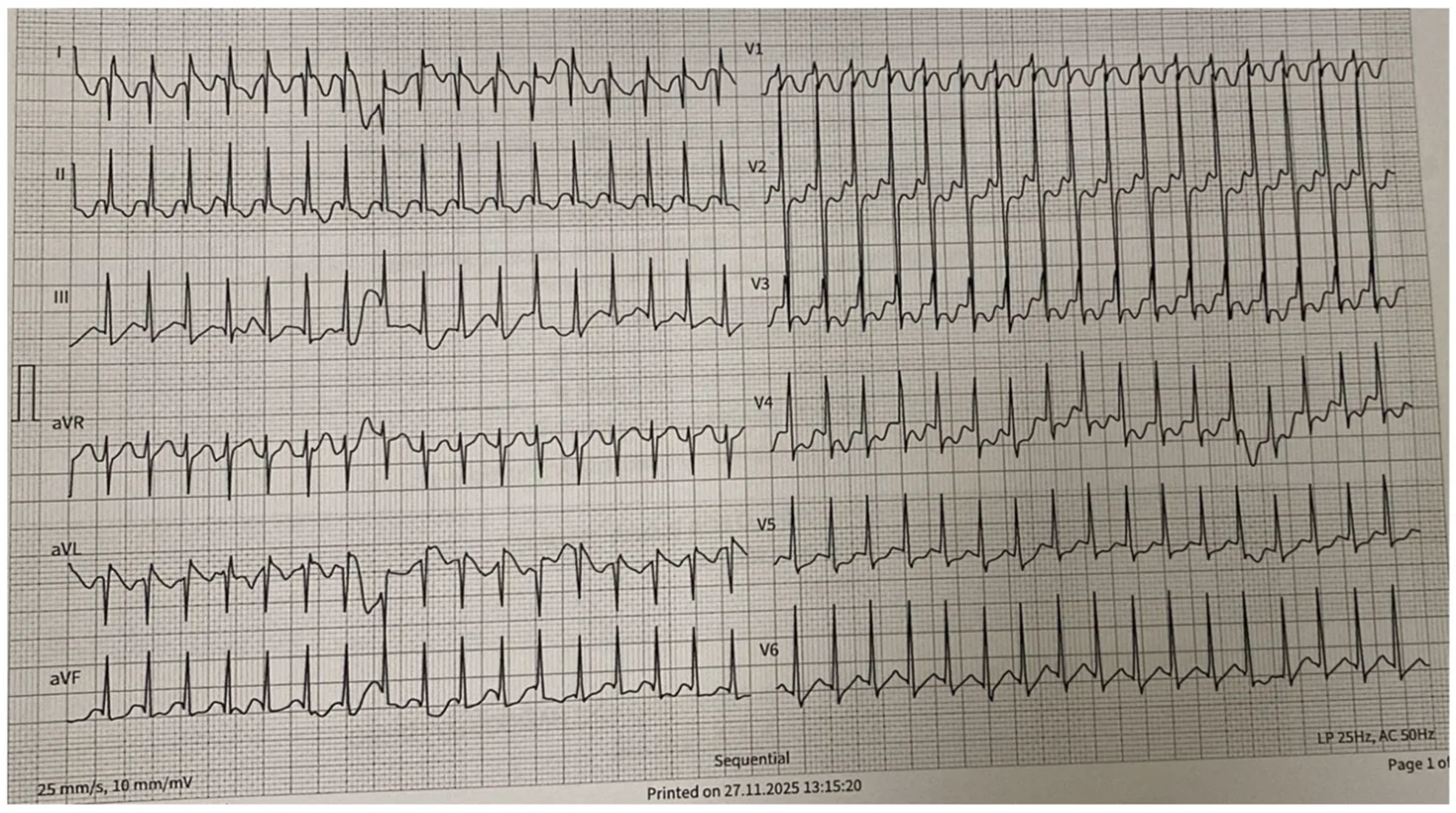
Representative electrocardiogram recorded during supraventricular tachycardia (SVT) in a patient with PLSVC who re-presented one week after the initial diagnosis.

One patient in the PLSVC group re-presented to the outpatient clinic with supraventricular tachycardia (SVT) one week after the initial diagnosis (Figure 2).

### 3.6. Sensitivity Analysis

After exclusion of the four PLSVC patients with low atrial rhythm, 22 PLSVC patients were compared with 26 controls. The overall pattern of electrocardiographic findings remained consistent with the primary analysis. P-wave dispersion, QTc dispersion, Tp–e dispersion, Tp–e/QT, QT/QRS, and QTc/QRS remained significantly higher in the PLSVC group, while Tp–e/QTc remained non-significant. These findings indicate that the principal ECG differences were not driven by the four patients with low atrial rhythm (Supplementary Table S2).

### 3.7. Observer Reliability

The second observer remeasured all 52 original ECGs independently while blinded to the first observer’s measurements. The first observer remeasured a random subset of 16 ECGs (8 PLSVC and 8 controls) at a separate time. Across 13 continuous ECG parameters, interobserver ICC(2,1) values ranged from 0.986 to 1.000, and intraobserver ICC(A,1) values ranged from 0.990 to 1.000; the lowest lower bound of the 95% CIs was 0.969. These findings indicated excellent interobserver and intraobserver reliability. Rhythm classification showed 100% interobserver agreement (Cohen’s kappa = 1.00). All 16 ECGs in the intraobserver subset were classified as sinus rhythm at both assessments; therefore, kappa was not estimable despite 100% raw agreement (Supplementary Table S3).

### 3.8. Correlations Between Coronary Sinus Measurements and Electrocardiographic Parameters

Correlations between coronary sinus measurements, age, and electrocardiographic parameters are summarized in Table 5.

Age showed significant correlations with coronary sinus dimensions. Age was positively correlated with CS diameter measured in the apical four-chamber view (r = 0.540, *p* = 0.004) and with CS diameter measured in the parasternal long-axis view (r = 0.527, *p* = 0.006). In contrast, age showed negative correlations with indexed CS diameter in the apical four-chamber view (r = −0.746, *p* < 0.001) and indexed CS diameter in the parasternal long-axis view (r = −0.779, *p* < 0.001).

No significant correlations were observed between coronary sinus measurements and P-wave dispersion or QTc dispersion (all *p* > 0.05).

Tp–e dispersion showed a significant negative correlation with indexed CS diameter measured in the parasternal long-axis view (r = −0.390, *p* = 0.049). Similarly, the Tp–e/QTc ratio demonstrated a significant negative correlation with indexed CS diameter in the parasternal long-axis view (r = −0.393, *p* = 0.047). No other significant correlations were observed between coronary sinus measurements and these parameters.

The QT/QRS ratio showed a significant negative correlation with CS diameter measured in the parasternal long-axis view (r = −0.439, *p* = 0.025). The QTc/QRS ratio demonstrated negative correlations with CS diameter measured in the apical four-chamber view (r = −0.415, *p* = 0.035) and in the parasternal long-axis view (r = −0.538, *p* = 0.005), whereas a positive correlation was observed with indexed CS diameter measured in the parasternal long-axis view (r = 0.402, *p* = 0.042).

## 4. Discussion

In this study, we demonstrated that arrhythmia-related electrocardiographic parameters were significantly increased in children with PLSVC. The significantly higher values of P-wave dispersion, QTc dispersion, and Tp–e dispersion—reflecting both atrial conduction and ventricular repolarization heterogeneity—in the PLSVC group suggest the presence of subclinical electrophysiological alterations in this population. In addition, the significantly increased indices of cardiac electrophysiological balance, namely the QT/QRS and QTc/QRS ratios, further support the presence of alterations in ventricular depolarization–repolarization balance.

Previous studies have suggested that PLSVC may be associated with an increased risk of atrial arrhythmias and conduction abnormalities. In particular, invasive electrophysiological investigations have demonstrated that PLSVC can serve as a source of ectopic atrial activity and non-pulmonary vein triggers, especially in patients with atrial fibrillation [4,5]. Similarly, persistent venous structures such as PLSVC may exhibit arrhythmogenic potential due to their unique anatomical and electrophysiological properties, including the presence of myocardial sleeves and abnormal conduction pathways [6].

In this context, our findings are consistent with the existing literature, suggesting that PLSVC is not merely a benign anatomical variant but may be associated with measurable alterations in cardiac electrophysiology. However, unlike previous studies that primarily focused on adult populations undergoing invasive evaluation, our study provides noninvasive evidence of electrophysiological alterations in a pediatric population with isolated PLSVC. This distinction is particularly important, as it suggests that such alterations may be present even in structurally normal hearts and in the absence of clinically overt arrhythmias.

In contrast, coronary sinus measurements demonstrated weak to moderate correlations with selected electrocardiographic parameters; however, these associations were not consistent across all indices, which is in line with previous studies suggesting that structural parameters alone may not fully explain electrophysiological variability in PLSVC [6]. While certain repolarization-related parameters, particularly QT/QRS and QTc/QRS, showed statistically significant correlations, most atrial conduction markers and several ventricular repolarization indices were not associated with coronary sinus size. In addition, only a limited number of parameters, such as Tp–e dispersion and Tp–e/QTc, showed significant correlations specifically with indexed coronary sinus measurements in the parasternal long-axis view. Moreover, the direction and strength of these correlations varied across different measurement methods (A4C vs. PLAX), and some significant associations were inverse in direction. These inverse and inconsistent relationships do not support a simple linear relationship in which greater coronary sinus enlargement is necessarily accompanied by greater electrical heterogeneity. Given the relatively small sample size of the PLSVC group, the observed bivariate correlations may also be sensitive to individual observations and should therefore be interpreted cautiously. Taken together, these findings suggest that coronary sinus size alone does not adequately explain the observed electrophysiological alterations and that coronary sinus dilatation may represent only one component of a multifactorial process.

Electrocardiographic alterations observed in our study are in line with previous reports evaluating atrial activation patterns in patients with PLSVC. Ito-Hagiwara et al. demonstrated that PLSVC is associated with distinct changes in P-wave morphology and frontal P-wave axis, reflecting altered atrial activation due to left-sided venous drainage [2]. Although their study primarily focused on atrial depolarization patterns, our findings extend these observations by demonstrating increased P-wave dispersion, a well-established marker of atrial conduction heterogeneity and arrhythmic risk [7,8].

In addition to atrial conduction abnormalities, we observed significant increases in QTc dispersion and Tp–e dispersion, which are recognized markers of ventricular repolarization heterogeneity and susceptibility to ventricular arrhythmias [9,10,11,12,13]. Increased Tp–e interval and its derived indices have been associated with a higher risk of malignant ventricular arrhythmias in various clinical conditions [11,12,13]. Furthermore, the significantly higher QT/QRS and QTc/QRS ratios in the PLSVC group suggest an imbalance between depolarization and repolarization processes, as reflected by indices of cardiac electrophysiological balance (ICEB and ICEBc). These indices have recently emerged as novel markers of arrhythmic risk and ventricular electrical instability [14,15].

The underlying mechanisms of the electrophysiological alterations observed in patients with isolated PLSVC are likely multifactorial. From an embryological perspective, PLSVC results from the persistence of the left cardinal venous system and is closely related to the development of the cardiac conduction system. During normal cardiac development, primitive pacemaker tissues are located near the venous inflow regions, and remnants of these structures may persist in association with PLSVC. This may contribute to abnormal automaticity and altered conduction properties [6].

While these developmental and anatomical mechanisms provide a plausible explanation for atrial conduction abnormalities, the mechanisms underlying the ventricular repolarization findings are less straightforward. Previous reports have suggested that marked coronary sinus dilatation may exert mechanical effects on adjacent atrioventricular structures, including stretching of the atrioventricular node and His bundle [6,16]. This may provide a potential anatomical link between PLSVC and electrical alterations extending beyond the atria. Therefore, mechanical effects related to coronary sinus dilatation may represent one possible mechanism contributing to the observed ventricular repolarization heterogeneity, potentially together with autonomic, neurohormonal, or other unmeasured factors; however, the present study was not designed to establish the underlying causal mechanism.

In addition, the close anatomical relationship between the coronary sinus and the adjacent atrial myocardium, along with the presence of myocardial sleeves, may facilitate the development of ectopic electrical activity. Invasive electrophysiological studies have demonstrated that PLSVC can serve as a source of non-pulmonary vein triggers and focal atrial arrhythmias [4,5]. Similarly, case-based electrophysiological observations have shown that PLSVC may act as a substrate for both reentrant and focal arrhythmias [17].

Taken together, these findings suggest that the electrophysiological alterations observed in our study may be related not only to secondary effects such as coronary sinus dilatation, but also to the intrinsic properties of the persistent venous structure. In our cohort, one 6-year-old patient developed a regular narrow-complex supraventricular tachycardia following the diagnosis of PLSVC, which was successfully terminated with a single dose of adenosine. Propranolol therapy (2 mg/kg/day in three divided doses) was initiated, and the patient has remained clinically controlled on medical therapy. Given the patient’s young age and satisfactory response to medical treatment, an electrophysiological study and catheter ablation were not performed at this stage, and electrophysiological evaluation is planned during follow-up. Consequently, the specific arrhythmia substrate could not be definitively established.

This study has several limitations that should be acknowledged. The relatively small sample size, along with the single-center and retrospective design, may limit the generalizability of the findings. Although the study had adequate power to detect large between-group differences, smaller effects may have remained undetected; therefore, non-significant findings should be interpreted cautiously. Bazett’s formula may overcorrect QTc at higher heart rates, which is an important consideration in pediatric populations with relatively high and variable heart rates [18]. Although heart rates were comparable between the PLSVC and control groups in the present study, potential heart rate-related overcorrection cannot be completely excluded. In addition, Holter monitoring was not systematically available, which may have limited the detection of intermittent or asymptomatic arrhythmic events. A validated severity grading system for coronary sinus dilatation was not available; therefore, coronary sinus enlargement was assessed using absolute and body surface area (BSA)-indexed dimensions rather than a standardized severity score. Furthermore, the occurrence of a single post-diagnostic SVT event cannot be used to estimate the true incidence of arrhythmias in children with isolated PLSVC. Long-term clinical follow-up was not systematically available, limiting assessment of the prognostic significance of the observed electrocardiographic abnormalities and their relationship with subsequent arrhythmic events. Finally, the absence of invasive electrophysiological evaluation precluded direct confirmation of the underlying mechanisms.

Despite these limitations, our study has several important strengths. To our knowledge, this is one of the first studies to comprehensively evaluate both atrial and ventricular electrocardiographic parameters, including indices of cardiac electrophysiological balance, in a pediatric population with isolated PLSVC. The inclusion of a well-matched control group enhances the validity of our comparisons. Furthermore, the preservation of the principal ECG findings after exclusion of patients with low atrial rhythm, together with the excellent interobserver and intraobserver reliability, supports the robustness of the main findings. Finally, the combined assessment of atrial conduction, ventricular repolarization, and coronary sinus morphology allowed these electrophysiological and anatomical features to be evaluated within the same pediatric PLSVC cohort.

## 5. Conclusions

In conclusion, children with isolated PLSVC exhibit significant alterations in electrocardiographic parameters reflecting both atrial conduction and ventricular repolarization heterogeneity. These findings suggest that isolated PLSVC may be associated with subclinical electrophysiological alterations involving both atrial conduction and ventricular repolarization. Importantly, the weak and inconsistent associations between coronary sinus dimensions and these electrocardiographic parameters indicate that the underlying mechanisms remain unclear and cannot be explained solely by coronary sinus dilatation. Further prospective studies involving larger cohorts, long-term clinical follow-up, and rhythm monitoring are needed to determine the clinical significance of these findings and their potential association with arrhythmic risk.

## Figures and Tables

**Table 1:** Demographic and clinical characteristics of the study population.

Variable	PLSVC Group (*n* = 26) Mean ± SD/Median (Min–Max)	Control Group (*n* = 26) Mean ± SD/Median (Min–Max)	PLSVC − Control Estimate (95% CI)	Effect Size	*p*-Value
Age (years)	9.37 ± 5.10/8.8 (0.4–17.8)	9.27 ± 4.95/9.0 (0.5–17.7)	0.10 (−2.70 to 2.90)	g = 0.02	0.943
Sex, *n* (%)	—	—	0.0 pp (−25.1 to 25.1)	RD = 0.000	1.000
Female	15 (57.7)	15 (57.7)	—	—	—
Male	11 (42.3)	11 (42.3)	—	—	—
Height (cm)	131.19 ± 29.97/132.0 (66–182)	130.69 ± 29.52/131.5 (68–185)	0.50 (−16.07 to 17.07)	g = 0.02	0.952
Body surface area (m^2^)	1.06 ± 0.41/1.05 (0.38–1.83)	1.06 ± 0.39/1.05 (0.40–1.82)	0.002 (−0.220 to 0.225)	g = 0.01	0.983
Weight (kg)	32.48 ± 17.40/29.50 (7.5–73.0)	32.15 ± 16.97/29.50 (8.0–72.0)	0.50 (−9.00 to 9.70)	δ = 0.02	0.905
Height Z-score	−0.34 ± 1.11/−0.35 (−1.78–1.83)	−0.37 ± 1.19/−0.44 (−1.95–1.83)	0.05 (−0.62 to 0.61)	δ = 0.02	0.891
Weight Z-score	−0.51 ± 1.01/−0.66 (−1.81–1.28)	−0.49 ± 1.00/−0.72 (−1.84–1.88)	−0.01 (−0.51 to 0.55)	δ = 0.00	0.985
Initial presentation, *n* (%)	—	—	—	—	—
Asymptomatic/incidental	18 (69.2)	—	—	—	—
Nonspecific chest pain	4 (15.4)	—	—	—	—
Arrhythmia-related presentation	4 (15.4)	—	—	—	—

Data are presented as mean ± SD and median (minimum–maximum), or *n* (%). Original SPSS *p* values are shown unchanged. Estimates are PLSVC minus control. For *t*-test comparisons, mean differences with 95% CIs and Hedges’ g are reported; for Mann–Whitney U comparisons, Hodges-Lehmann shifts with 10,000-resample bootstrap 95% CIs (seed = 20260810) and Cliff’s delta are reported. RD: risk difference; pp: percentage points. —, not applicable.

**Table 2:** Comparison of conventional echocardiographic parameters between patients with PLSVC and healthy controls.

Variable	PLSVC Group (*n* = 26) Mean ± SD/Median (Min–Max)	Control Group (*n* = 26) Mean ± SD/Median (Min–Max)	PLSVC − Control Estimate (95% CI)	Effect Size	*p*-Value
**IVSD (mm)**	6.28 ± 1.29/6.0 (4.5–10.4)	6.28 ± 1.08/6.45 (4.0–8.0)	−0.10 (−0.80 to 0.50)	δ = −0.07	0.653
**LVDD (mm)**	39.11 ± 7.71/38.25 (24–53)	38.73 ± 6.95/39.0 (27–52)	0.38 (−3.71 to 4.47)	g = 0.05	0.852
**LVSD (mm)**	24.10 ± 4.55/24.0 (15–35)	24.50 ± 5.00/25.0 (17–34)	−0.40 (−3.06 to 2.26)	g = −0.08	0.764
**LVPWD (mm)**	5.89 ± 1.31/5.65 (4–9)	5.62 ± 0.96/5.80 (4–7)	0.27 (−0.37 to 0.90)	g = 0.23	0.408
**IVSD Z-score**	−0.96 ± 0.85/−1.32 (−1.98–1.00)	−0.94 ± 0.75/−1.09 (−1.94–0.28)	−0.04 (−0.47 to 0.37)	δ = −0.04	0.819
**LVDD Z-score**	−0.26 ± 1.26/−0.61 (−1.95–1.58)	−0.36 ± 0.77/−0.30 (−1.67–1.08)	−0.03 (−0.68 to 0.76)	δ = −0.01	0.956
**LVSD Z-score**	−0.51 ± 1.14/−0.76 (−1.91–1.76)	−0.40 ± 0.92/−0.57 (−1.93–1.20)	−0.17 (−0.76 to 0.42)	δ = −0.10	0.546
**LVPWD Z-score**	−1.11 ± 0.85/−1.33 (−1.98–0.92)	−1.35 ± 0.48/−1.44 (−1.85–0.34)	0.05 (−0.19 to 0.50)	δ = 0.06	0.694
**EF (%)**	68.62 ± 4.80/68 (62–76)	67.58 ± 5.46/67 (60–79)	1.04 (−1.82 to 3.90)	g = 0.20	0.470
**FS (%)**	37.50 ± 4.62/35.5 (30–45)	37.15 ± 4.14/36.5 (32–47)	0.00 (−2.00 to 3.00)	δ = 0.05	0.733

Data are presented as mean ± SD and median (minimum–maximum). Original SPSS *p* values are shown unchanged. Estimates are PLSVC minus control. IVSD: interventricular septal thickness; LVDD: left ventricular end-diastolic diameter; LVSD: left ventricular end-systolic diameter; LVPWD: left ventricular posterior wall thickness; EF: ejection fraction; FS: fractional shortening.

**Table 3:** Comparison of coronary sinus dimensions between patients with PLSVC and healthy controls.

Variable	PLSVC Group (*n* = 26) Mean ± SD/Median (Min–Max)	Control Group (*n* = 26) Mean ± SD/Median (Min–Max)	PLSVC − Control Estimate (95% CI)	Effect Size	*p*-Value
CS diameter (mm), A4C	9.01 ± 2.30/8.75 (4.5–13.3)	3.42 ± 0.99/3.30 (2.0–5.0)	5.30 (4.45 to 6.40)	δ = 0.99	<0.001
Indexed CS diameter (mm/m^2^), A4C	9.50 ± 3.39/8.97 (4.10–18.60)	3.57 ± 1.29/3.54 (1.39–6.75)	5.51 (4.29 to 7.17)	δ = 0.95	<0.001
CS diameter (mm), PLAX	8.73 ± 2.12/8.20 (5.10–13.30)	—	—	—	—
Indexed CS diameter (mm/m^2^), PLAX	9.26 ± 3.31/8.54 (3.88–15.40)	—	—	—	—

Data are presented as mean ± SD and median (minimum–maximum). Original SPSS *p* values are shown unchanged. PLAX coronary sinus measurements were obtained only in the PLSVC group, as the coronary sinus could not be consistently visualized or measured in the control group; therefore, these measurements are presented descriptively. CS: coronary sinus; A4C: apical four-chamber view; PLAX: parasternal long-axis view. —, not applicable.

**Table 4:** Comparison of electrocardiographic parameters between patients with PLSVC and healthy controls.

Variable	PLSVC Group (*n* = 26) Mean ± SD/Median (Min–Max)	Control Group (*n* = 26) Mean ± SD/Median (Min–Max)	PLSVC − Control Estimate (95% CI)	Effect Size	*p*-Value
Heart rate (bpm)	98.69 ± 25.32/95.5 (56–161)	93.15 ± 20.71/91 (60–139)	5.54 (−7.34 to 18.42)	g = 0.24	0.392
Rhythm, *n* (%)	—	—	15.4 pp (−0.5 to 33.5)	RD = 0.154	0.110
Sinus rhythm	22 (84.6)	26(100.0)	—	—	—
Low atrial rhythm	4 (15.4)	0(0.0)	—	—	—
P-wave axis (°)	29.72 ± 24.22/32 (−20–71)	37.39 ± 14.24/38.5 (7–67)	−7.67 (−18.80 to 3.46)	g = −0.38	0.172
QRS axis (°)	53.79 ± 28.46/60 (0–130)	58.35 ± 15.58/60.5 (30–84)	−3.00 (−16.00 to 8.51)	δ = −0.11	0.498
T-wave axis (°)	38.89 ± 24.59/39.5 (9–130)	40.50 ± 12.46/41.5 (17–67)	−2.00 (−14.00 to 5.00)	δ = −0.11	0.498
P-wave dispersion (ms)	62.15 ± 14.78/57.14 (36–100)	35.59 ± 10.02/35.30 (22.22–70.59)	23.81 (20.64 to 33.61)	δ = 0.91	<0.001
QTc dispersion (ms)	99.66 ± 30.10/99.40 (51.91–167.76)	40.45 ± 10.78/40.34 (22.98–61.68)	58.47 (44.41 to 71.24)	δ = 0.97	<0.001
Tp–e dispersion (ms)	67.29 ± 19.10/62.67 (40–114.28)	38.42 ± 7.13/35.60 (22.22–55.56)	25.57 (18.89 to 36.25)	δ = 0.89	<0.001
Tp–e/QT	0.237 ± 0.029/0.234 (0.190–0.291)	0.218 ± 0.024/0.218 (0.176–0.270)	0.019 (0.004 to 0.034)	g = 0.70	0.013
Tp–e/QTc	0.189 ± 0.027/0.182 (0.140–0.240)	0.181 ± 0.022/0.178 (0.151–0.230)	0.009 (−0.005 to 0.023)	g = 0.36	0.195
QT/QRS	4.33 ± 0.59/4.29 (3.25–5.50)	3.78 ± 0.36/3.77 (3.22–4.62)	0.540 (0.230 to 0.855)	δ = 0.54	<0.001
QTc/QRS	5.48 ± 0.91/5.58 (3.55–7.23)	4.61 ± 0.36/4.64 (3.61–5.23)	0.875 (0.380 to 1.340)	δ = 0.62	<0.001

Data are presented as mean ± SD and median (minimum–maximum), or *n* (%). Original SPSS *p* values are shown unchanged. For continuous variables, estimates and effect sizes follow the definitions in Table 1. For rhythm, the estimate is the risk difference for low atrial rhythm with a Newcombe 95% CI. QTc: corrected QT interval; Tp–e: T-peak to T-end interval. —, not applicable.

**Table 5:** Correlations between coronary sinus measurements and electrocardiographic parameters.

Variable	CS Diameter (mm), A4C r (p)	CS Index (mm/m^2^), A4C r (p)	CS Diameter (mm), PLAX r (p)	CS Index (mm/m^2^), PLAX r (p)
Age	0.540 (0.004)	−0.746 (<0.001)	0.527 (0.006)	−0.779 (<0.001)
P-wave dispersion	−0.077 (0.709)	−0.187 (0.359)	−0.050 (0.807)	−0.138 (0.502)
QTc dispersion	−0.149 (0.468)	0.268 (0.186)	−0.309 (0.124)	0.202 (0.322)
Tp–e dispersion	−0.051 (0.805)	−0.330 (0.099)	−0.181 (0.375)	−0.390 (0.049)
Tp–e/QT	−0.111 (0.589)	0.168 (0.412)	−0.014 (0.946)	0.141 (0.491)
Tp–e/QTc	0.138 (0.501)	−0.359 (0.072)	0.257 (0.205)	−0.393 (0.047)
QT/QRS	−0.365 (0.067)	0.003 (0.989)	−0.439 (0.025)	−0.015 (0.943)
QTc/QRS	−0.415 (0.035)	0.415 (0.035)	−0.538 (0.005)	0.402 (0.042)

Values are Spearman correlation coefficients (r), with two-sided *p* values in parentheses. The original correlation coefficients and *p* values are reproduced unchanged. CS: coronary sinus; A4C: apical four-chamber view; PLAX: parasternal long-axis view; QTc: corrected QT interval; Tp–e: T-peak to T-end interval.

## Data Availability

The data supporting the findings of this study are available from the corresponding author upon reasonable request, in accordance with institutional policies and patient confidentiality.

## Supplementary Materials

The following supporting information can be downloaded at: https://www.mdpi.com/article/10.3390/jcm15166494/s1, Table S1: Fixed-sample sensitivity power analysis for *n* = 26 PLSVC patients and *n* = 26 controls; Table S2: Sensitivity analysis after exclusion of four PLSVC patients with low atrial rhythm; Table S3: Interobserver and intraobserver reliability of continuous ECG measurements.
