# Supplementary material for: Electrophysiological Changes in Children with Isolated Persistent Left Superior Vena Cava: A Case–Control Study
Source: J Clin Med. 2026 Aug 21;15(16):6494. doi: 10.3390/jcm15166494 (PMC13514265; doi:10.3390/jcm15166494)
Supplement: Supplementary file 1 [file jcm-15-06494-s001.zip › jcm-4297786-supplementary.pdf]

Supplementary Table S1. Fixed-sample sensitivity power analysis for n = 26 PLSVC patients and n = 26 controls

Scenario	Analysis framework	Result
**Assumed effect d = 0.20**	Two-sided independent-samples t-test; α = 0.05; n = 26 + 26	Power = 10.9%
**Assumed effect d = 0.50**	Two-sided independent-samples t-test; α = 0.05; n = 26 + 26	Power = 42.4%
**Assumed effect d = 0.80**	Two-sided independent-samples t-test; α = 0.05; n = 26 + 26	Power = 80.7%
**Assumed effect d = 1.00**	Two-sided independent-samples t-test; α = 0.05; n = 26 + 26	Power = 94.2%
**Target power = 80%**	Two-sided α = 0.05; n = 26 + 26	Minimum detectable effect d = 0.792
**Target power = 90%**	Two-sided α = 0.05; n = 26 + 26	Minimum detectable effect d = 0.917
**Target power = 95%**	Two-sided α = 0.05; n = 26 + 26	Minimum detectable effect d = 1.020

Two-sided independent-samples t-test framework with alpha = 0.05. This is a fixed-sample sensitivity analysis and is not an observed post hoc power calculation based on obtained p values.

Supplementary Table S2. Sensitivity analysis after exclusion of four PLSVC patients with low atrial rhythm

ECG variable	PLSVC (n = 22)	Control (n = 26)	PLSVC − control estimate (95% CI)	Effect size	p-value
**Heart rate (bpm)**	99.95 ± 24.78	93.15 ± 20.71	6.80 (−6.66 to 20.26)	g = 0.30	0.313
**P-wave axis (°)**	37.44 ± 16.92	37.38 ± 14.24	0.05 (−9.16 to 9.26)	g = 0.00	0.991
**QRS axis (°)**	55.55 [27.43–70.00]	60.50 [49.25–70.50]	−5.45 (−20.00 to 6.40)	δ = −0.18	0.286
**T-wave axis (°)**	39.50 [22.40–46.75]	41.50 [32.75–46.75]	−2.00 (−13.00 to 5.00)	δ = −0.12	0.494
**P-wave dispersion (ms)**	57.14 [57.14–71.43]	35.30 [32.02–40.49]	24.68 (21.25 to 34.92)	δ = 0.91	<0.001
**QTc dispersion (ms)**	100.89 [85.06–114.84]	40.34 [31.32–49.32]	61.63 (48.43 to 74.77)	δ = 0.99	<0.001
**Tp-e dispersion (ms)**	68.39 [57.14–81.71]	35.60 [34.47–44.32]	27.06 (18.78 to 37.78)	δ = 0.87	<0.001
**Tp-e/QT**	0.24 ± 0.03	0.22 ± 0.02	0.024 (0.009 to 0.040)	g = 0.92	0.003
**Tp-e/QTc**	0.19 ± 0.02	0.18 ± 0.02	0.011 (−0.003 to 0.024)	g = 0.46	0.119
**QT/QRS**	4.39 [4.00–4.94]	3.77 [3.62–3.94]	0.570 (0.260 to 0.920)	δ = 0.59	<0.001
**QTc/QRS**	5.58 [4.89–6.21]	4.64 [4.40–4.87]	0.935 (0.408 to 1.390)	δ = 0.70	<0.001

Four PLSVC patients with low atrial rhythm were excluded. To isolate the effect of case exclusion and maintain direct comparability with Table 4, the same variable-specific parametric or non-parametric analysis family, effect estimate, and effect-size metric used in Table 4 were retained. Variables analysed with a t test are shown as mean ± SD; variables analysed with the Mann–Whitney U test are shown as median [interquartile range]. Hodges–Lehmann bootstrap CIs used 10,000 resamples with seed = 20260810.

Supplementary Table S3. Interobserver and intraobserver reliability of continuous ECG measurements

Parameter	Inter-observer ICC(2,1) (95% CI), n = 52	Intra-observer ICC(A,1) (95% CI), n = 16	Interpretation
**PR interval (ms)**	0.998 (0.996–0.999)	0.997 (0.969–0.999)	Excellent
**P-wave duration (ms)**	1.000 (1.000–1.000)	0.998 (0.994–0.999)	Excellent
**P-wave dispersion (ms)**	1.000 (1.000–1.000)	0.999 (0.996–1.000)	Excellent
**QRS duration (ms)**	0.999 (0.994–1.000)	0.999 (0.997–1.000)	Excellent
**QT interval (ms)**	1.000 (1.000–1.000)	1.000 (1.000–1.000)	Excellent
**QTc interval (ms)**	0.986 (0.975–0.992)	1.000 (1.000–1.000)	Excellent
**QTc dispersion (ms)**	1.000 (1.000–1.000)	0.998 (0.995–0.999)	Excellent
**Tp-e interval (ms)**	0.998 (0.974–0.999)	0.994 (0.982–0.998)	Excellent
**Tp-e dispersion (ms)**	1.000 (0.996–1.000)	0.999 (0.998–1.000)	Excellent
**Tp-e/QT**	0.993 (0.987–0.996)	0.990 (0.970–0.997)	Excellent
**Tp-e/QTc**	0.994 (0.989–0.997)	0.995 (0.985–0.998)	Excellent
**QT/QRS**	0.998 (0.990–0.999)	0.998 (0.993–0.999)	Excellent
**QTc/QRS**	0.994 (0.983–0.997)	0.999 (0.996–1.000)	Excellent

Interobserver reliability used a two-way random-effects, absolute-agreement, single-measure ICC(2,1) model for all 52 ECGs. Intraobserver reliability used a two-way mixed-effects, absolute-agreement, single-measure ICC(A,1) model for 16 randomly selected ECGs (8 PLSVC and 8 controls). The second observer was blinded to the first observer's measurements, and all measurements were performed independently from the original ECG records.
